# The Preparation of TiO_2_ Film by the Sol-Gel Method and Evaluation of Its Self-Cleaning Property

**DOI:** 10.3390/ma11030450

**Published:** 2018-03-19

**Authors:** Yu Liang, Sijia Sun, Tongrong Deng, Hao Ding, Wanting Chen, Ying Chen

**Affiliations:** 1 Beijing Key Laboratory of Materials Utilization of Nonmetallic Minerals and Solid Wastes, National Laboratory of Mineral Materials, School of Materials Science and Technology, China University of Geosciences (Beijing), Beijing 100083, China; liangyuaadd@126.com or liangyuaadd@gmail.com (Y.L.); ssjcugb@163.com (S.S.); 18010157480@163.com (T.D.); wantingchen123@163.com (W.C.); yingchen1827@163.com (Y.C.); 2 School of Materials Science and Technology, Shenyang University of Chemical Technology, Shenyang 110142, China

**Keywords:** sol-gel method, TiO_2_ film, hydrolysis control agent, anti-fogging property, self-cleaning property

## Abstract

TiO_2_ sol was produced by the sol-gel method through the hydrolysis and the aging of tetrabutyl titanate and the TiO_2_ film was obtained by dipping and uniform lifting of the acid-treated and ultrasound-treated clean glass slides into the TiO_2_ sol followed by aging, drying, and calcination. The effect of the hydrolysis control agents to the formed sol was researched and the crystalline state, the morphology, and the photocatalytic properties of the products after calcination were characterized. The structural morphology, the contact angles before and after illumination, and the self-cleaning properties of the TiO_2_ film were characterized as well. The results showed that by using acetylacetone as the hydrolysis control agent, the formed TiO_2_ sol had relatively high stability. The product after the calcination of the TiO_2_ sol was of single anatase type with crystalline size of 18–20 nm and it could degrade nearly 100% of methylene blue after 90 min illumination. The formed TiO_2_ film is compact, continuous, smooth, and had the properties of super-hydrophilicity (after 30 min illumination due to its contact angle decreasing from 21° to nearly 0°) and anti-fogging capability, which indicated its excellent self-cleaning property.

## 1. Introduction

Titanium dioxide is a kind of material that is mostly used as white pigments for its outstanding properties, such as high whiteness, brightness, non-toxicity, and strong hiding power [1]. It is also well known for its excellent chemical stability, biological nontoxicity, and low cost [2,3,4]. It has been used extensively as a photocatalyst since 1971, when articles published by Formenti et al. [5] and K. Honda [6] caused a global upsurge of the extensive use of TiO_2_ as a photocatalyst. Since this decade, a number of processes, such as effluent detoxification and disinfection, water splitting, and organic synsthesis, have used nano-TiO_2_ as photocatalysis [7,8,9,10,11,12]. A large amount of TiO_2_ has been used in Asia, especially in China, South Korea, and Japan [13,14,15]. The self-cleaning effect of TiO_2_ under ultraviolet (UV) irradiation due to the photoinduced hydrophilic properties of TiO_2_ is also a feature [16,17]. Clear and unified explanations have been made regarding the mechanisms of the TiO_2_ photocatalysis process. A series of reactions have been induced by the photogenerated e^−^ (electrons) and h^+^ (holes) from the photoexcited catalyst in the system. O$\begin{array}{c} -\\ 2 \end{array}$ and subsequent H_2_O_2_ and OH• radicals [18,19] were produced as a result of these different photoreactions and then facilitated the decomposition of the organic substances [20,21]. Two effects lead to the self-cleaning property of nano-TiO_2_ [22,23]. One is that the active components induced by the photocatalytic action of TiO_2_ can react with the pollutants adhering to the surface, thus achieving the decomposition of pollutants. The other is that the decomposed products can be washed away by rain, which maintains the cleanness of the material surface [24]. Kim et al. [23] synthesized a layer of water-resistant and surface-coatable C-PEG (poly(ethyleneglycol)-g-[poly(dimethylaminomethacrylate)]) with TiO_2_ nanoarticles (NPs) on a glass surface via catechol chemistry and they found that the final product gained self-cleaning capabilities owing to the hydrophilic nature imparted to the surface by controlling the amounts of catechol moiety and TiO_2_ NPs on the polymer. Lucas et al. [25] combined phase change materials (PCM) microcapsules and TiO_2_ nanoparticles together and found it made mortars with multiple functionalities including self-cleaning properties. Zhang et al. [26] produced a nano-TiO_2_ polyacrylate hybrid dispersion and successfully fixed it on cotton fabric. The final product was proved to have good self-cleaning properties by decomposing the red wine besmirch on it under natural light.

Various ways have been developed to synthesize nano-sized TiO_2_ particles or TiO_2_ film. Among them, the most important methods of producing titanium dioxide are the hydrolysis and calcination of titanium sulfate and the oxidation of titanium tetrachloride [27]. Other synthesis methods are precipitation of titanium hydroxide, pyrolysis of the titanium citrate precursor [28], and hydrolysis of TiCl_4_. The sol-gel method was first reported in 1939 by Geffcken and Berger and has received great attention since then. It has become a commonly used method for producing TiO_2_ due to its simple operation, low reaction temperature, good chemical homogeneity, low requirements for the substrates, and high purity of the product. However, some factors have restricted the scope of applications of nano-TiO_2_. TiO_2_ nanoparticles tend to form serious agglomerations, which of course increase the apparent grain size to the micron scale and inhibit its performance, including the self-cleaning effect. The recovery of nano-TiO_2_ particles is also a problem. It is hard to recover nano-TiO_2_ powder from water, thus forming secondary pollution. The residual nano-TiO_2_ particles can restrain the growth and reproduction of cells, produce free radicals, and harm DNA [29,30]. In order to solve the problem of recycling TiO_2_, it is beneficial to produce TiO_2_ film instead of producing TiO_2_ nanoparticles. Barbana et al. [31] produced TiO_2_ film using the technique of pulsed electrophoretic deposition on the surface of stainless steel and found that the TiO_2_ film exhibited good photocatalytic activity due to its crystalline size and the synergetic effect of Fe_2_O_3_. However, there are few articles talking about the effects of the main parameters and their optimization during the preparation process of TiO_2_ film. Among them, TiO_2_ sol is the precursor of TiO_2_ film and has extremely important impacts on the formed film. On the one hand, the stability of the sol has an extremely important role in the uniformity of the formed TiO_2_ film. Only transparent and precipitation-free sol can be used to produce TiO_2_ film successfully. On the other hand, the crystalline phase of the sol after calcination should be in single anatase phase or in anatase phase with a slight amount of rutile phase with its crystal size in the nano-scale, thus leading to good photocatalytic degradation ability and light-induced hydrophilic effect of the formed TiO_2_ thin film.

In this article, the TiO_2_ sol and TiO_2_ film were produced by the hydrolysis of tetrabutyl titanate under three different hydrolysis control agents by the sol-gel method. The effects of the hydrolysis control agents, the structures of the particles after the calcination of the sol, and the self-cleaning properties of the TiO_2_ film were investigated and evaluated.

## 2. Materials and Methods

### 2.1. Materials

Tetrabutyl titanate was obtained from TCI company in Shanghai (China). Acetylacetone and acetic acid were purchased from Xilong Chemical Co., Ltd. in Guangdong Province (Dongguan, China). Anhydrous ethanol, triethanolamine, hydrochloric acid, and methylene blue were obtained from Beijing Chemical Plants in Beijing (China). They were all analytical agents. All chemicals were used as received without further purification.

### 2.2. Preparation of TiO_2_ Powder and TiO_2_ Thin Film

Tetrabutyl titanate and the hydrolysis control agent were dissolved in anhydrous ethanol separately and stirred adequately. Then, the hydrolysis control agent solution was added into the tetrabutyl titanate solution and stirred adequately. The mixed solution of water and anhydrous ethanol were added to the former mixed solution drop by drop with magnetic stirrer stirring for 12 h in room temperature, forming TiO_2_ sol. The mole ratio of tetrabutyl titanate to hydrolysis control agent to water was 1:0.5:2. The TiO_2_ powder was obtained by aging treatment, drying (at 60 °C), and calcination of TiO_2_ sol. The TiO_2_ thin film was obtained by dipping and uniformly lifting the acid-treated and ultrasound-treated clean glass slides into the TiO_2_ sol followed by aging, drying, and calcination.

### 2.3. Characterization

The X-ray diffraction patterns were obtained on a Rigaku Rotaflex X-ray powder diffractometer (Rigaku, Tokyo, Japan), employing Cu Kα radiation, 40 kV, 100 mA. The X-ray diffraction (XRD) patterns in the 2θ range from 3° to 70° were collected at 4°/min. The contact angle was measured by Rame-Hart 200-F1 goniometer (Rame-hurt, Succasunna, NJ, USA) using deionized water as the media. The UV-vis diffuse reflectance spectra were tested with a spectrophotometer (Cary 5000, Varian, Palo Alto, CA, USA). The microstructure and morphologies were investigated by scanning electron microscopy (SEM; S-3500N, Hitachi, Tokyo, Japan) and transmission electron microscopy (TEM; JEOL JEM-2010, JEOL, Tokyo, Japan). The photocatalytic activities were evaluated by the decomposition of methylene blue under UV light illumination (λ < 420 nm). In a typical photocatalytic experiment, 50 mg samples were dispersed in 50 mL methylene blue solution (with its concentration 10 ppm) in a photochemical reactor (BL-GHX-V, Shanghai, China). Prior to illumination, the suspension was magnetically stirred in the dark for 1 h to achieve the adsorption–desorption equilibrium on the surface of the photocatalyst. Light illumination was conducted using a 300 W mercury lamp as the UV light resonance. During the photodecomposition process, samples were withdrawn at regular intervals and centrifuged to remove the photocatalyst for analysis. The filtrate was then analyzed using a UV-vis spectrophotometer to measure the absorption of methylene blue in the range of 200–800 nm. The self-cleaning property of the TiO_2_ film was tested by dropping methylene blue on the surfaces with or without TiO_2_ film. When the liquid dried, these surfaces were illuminated under UV light for 30 min and washed slowly with water. The self-cleaning property of the film was judged by the relative area of the remaining methylene blue. The antifogging property of TiO_2_ film was judged by illuminating the surfaces with or without TiO_2_ film for 30 min under UV light, then water was sprayed on the surfaces to see how many water droplets emerged on the surfaces.

## 3. Results and Discussion

### 3.1. The Effects of Different Hydrolysis Agents on the Stability and Crystalline Phase of TiO_2_ Sol

#### 3.1.1. The Stability of TiO_2_ Sol 

Three different hydrolysis control agents (acetylacetone, triethanolamine, acetic acid, and hydrochloric acid mixture (2.5:1, volume ratio)) were used in this study to prepare TiO_2_ sol. The mole ratio of tetrabutyl titanate to hydrolysis control agent to water was 1:0.5:2. The colors of the formed sol were observed and recorded (Table 1) (The sol is transparent, if not clearly mentioned “there is precipitation”). When the hydrolysis control agents added were acetylacetone and the mixture of acetic acid and hydrochloric acid, respectively, the color and status of the TiO_2_ sol can be maintained as long as 48 h, meaning good stability of the sol. However, when the hydrolysis control agent was triethanolamine, the sol was not that stable and formed some precipitation when the aging time was 48 h.

#### 3.1.2. The Crystalline Phase of TiO_2_ after Calcination of the Sol

**Figure 1:**
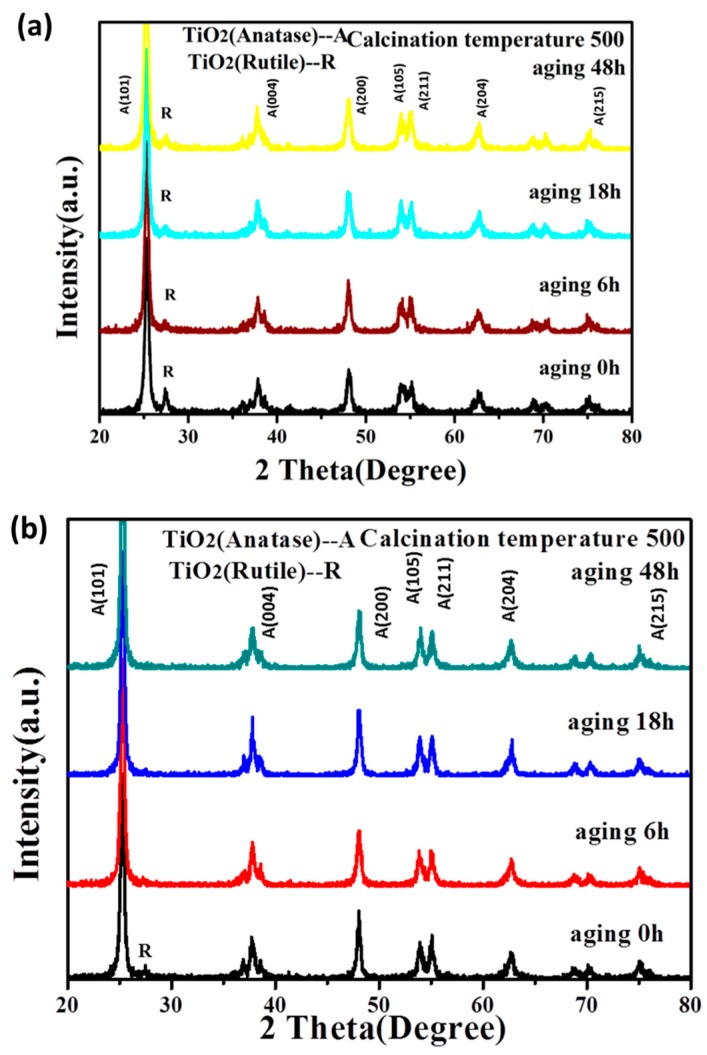

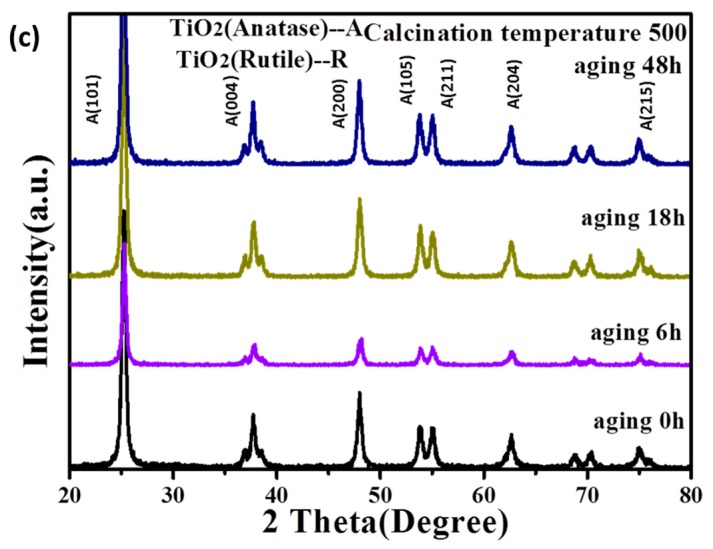
The XRD patterns of TiO_2_ powder formed by the calcination of the sol using different hydrolysis control agents with different aging time. The hydrolysis control agents were: (**a**) triethanolamine; (**b**) acetylacetone; (**c**) the mixture of acetic acid and hydrochloric acid. The calcination temperature was 500 °C.

XRD patterns of the products obtained by the calcination of the sol in different hydrolysis control agents and aging times are shown in Figure 1. Anatase TiO_2_ formed after calcination of the sol at 500 °C by all three hydrolysis control agents with some rutile TiO_2_ formed, together marked as R.

When the hydrolysis control agent was triethanolamine, rutile TiO_2_ powder formed together with anatase TiO_2_. Compared with others, the intensity of the characteristic peaks of the rutile TiO_2_ was the largest when the aging time was 0 h, corresponding to the highest amount of rutile TiO_2_. The amount of rutile phase decreased but still existed as the aging time was between 6 h and 48 h. Rutile phase TiO_2_ only existed in the product when using acetylacetone as the hydrolysis control agent and the aging time is 0 h. When the hydrolysis control agent was the mixture of acetic acid and hydrochloric acid, the formed TiO_2_ powder was in a single anatase type and did not change as the aging time increased.

Moreover, the crystal size of TiO_2_ at (101) was calculated by the Debye–Scherrer equation:

(1) $$D_{\mathrm{hkl}}=\frac{k\lambda }{\beta \cos ɵ}$$

where D_hkl_ is the average crystal size at (hkl) crystal plane; k is the form factor, which is close to 1; β is full width at half maximum (FWHM); λ is the wavelength of the x-ray; θ is the diffraction angle. The results showed that the anatase crystal size of TiO_2_ was 20–21 nm, 16–17 nm, and 18–19 nm when using acetylacetone, triethanolamine, and the mixture of acetic acid and hydrochloric acid as hydrolysis control agent, respectively. The crystal size of TiO_2_ after calcination was kept at a relatively small scale and was not affected by aging time.

Based on the above experimental results, it can be concluded that acetylacetone is the most suitable hydrolysis control agent for the formation of TiO_2_ sol. Triethanolamine is not that suitable, based on the facts that the sol formed was not stable and the crystalline type of the final products is not pure anatase type TiO_2_, but a mixture of rutile and anatase type TiO_2_. Considering the environmental effect, the mixture of acetic acid and hydrochloric acid is not as suitable as acetylacetone.

### 3.2. The Effect of Calcination Temperature to the Crystalline Phase and Morphology of TiO_2_

**Figure 2:**
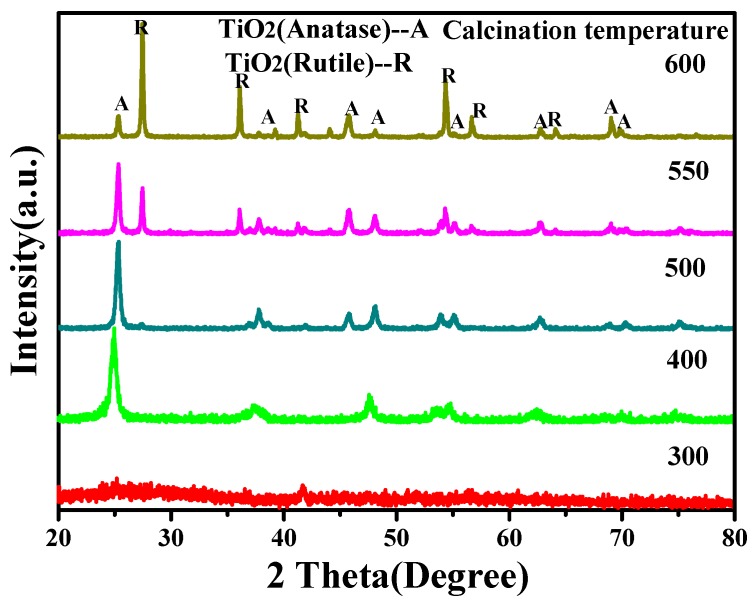
The XRD patterns of TiO_2_ powder after being calcined at 300 °C, 400 °C, 500 °C, 550 °C, and 600 °C. The hydrolysis agent was acetylacetone and the aging time was 6 h.

Figure 2 shows the XRD patterns of the samples obtained after TiO_2_ sol being calcined at different temperatures. When the calcination temperature was 300 °C, there was no obvious crystal formation, except for the broad scattering peak when 2θ was 25°. As the temperature increased to 400–500 °C, the diffraction peak of anatase TiO_2_ emerged with a slight amount of rutile TiO_2_. The higher the temperature, the sharper the peak shape. When the temperature increased to 550 °C, the final product was a mixture of anatase TiO_2_ and rutile TiO_2_. The diffraction peak intensity of rutile TiO_2_ increased further when the temperature reached 600 °C, which is in accordance with the literature [32]. Meanwhile, the peak intensity of anatase TiO_2_ decreased to a relatively low level. Since anatase TiO_2_ has much better photocatalytic properties than rutile TiO_2_, the proper temperature is 500 °C.

**Figure 3:**
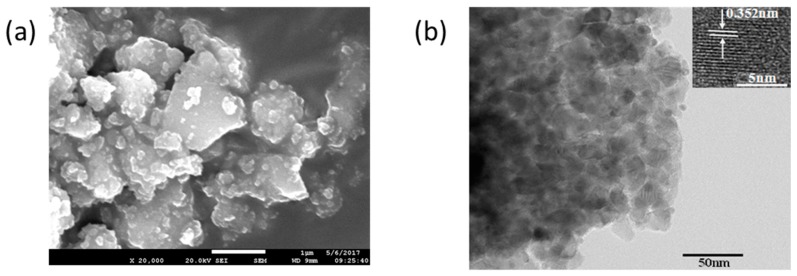
(**a**) SEM image of the TiO_2_ powders produced by using acetylacetone as hydrolysis control agent and calcined at 500 °C; (**b**) TEM image of the TiO_2_ powders produced by using acetylacetone as hydrolysis control agent with calcination temperature 500 °C.

From the SEM images of the samples produced after the calcination of TiO_2_ sol using acetylacetone as the hydrolysis control agent (Figure 3 and Supplementary Materials Figure S1), it can be seen that when the calcination temperature was relatively low (100 °C and 300 °C), the particles were large with maximum particle size of 3–5 µm and had poor dispersity. When the calcination temperature increased to 400 and 500 °C, the particle size decreased below 1 µm with good dispersity, which means stable TiO_2_ grains had formed. The grain size increased to 1–2 µm when the calcination temperature was 600 °C. From the TEM image (Figure 3b), it can be seen that its primary grain size was about 20 nm, which was in accordance with the XRD result. The enlarged images showed the interplanar spacing of the (101) face of anatase TiO_2_ to be 0.352 nm. Therefore, the micron size particles in the SEM images are only the aggregates of these nano-sized particles.

### 3.3. The Optical Properties and Photocatalytic Activity of TiO_2_ Obtained by the Calcination of TiO_2_ Sol

**Figure 4:**
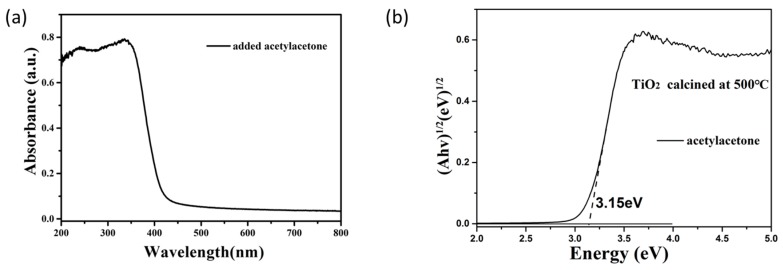
(**a**) UV-vis diffuse reflectance spectrum of TiO_2_ produced by using actylacetone as the hydrolysis control agent; (**b**) The plots between (αhν)^1/2^ and Eg for TiO_2_ with acetylacetone as the hydrolysis control agent.

Figure 4a displays the UV-visible diffuse reflectance spectrum in the wavelength range of 200–800 nm for TiO_2_ obtained by the calcination of the TiO_2_ sol. Its band-gap value was calculated using the UV-visible absorption spectrum according to the Kubelka–Munk equation [33]:

αh_ν_ = A (hν − Eg)^n/2^ (2)

where α is the adsorption coefficient; h_ν_ the photo energy; A the proportionality constant; Eg the band gap energy (eV). n equals to 1 or 4, depending on whether the transition is direct or indirect, respectively. Since the transition between the bands in TiO_2_ is indirect, n = 4. The energy of the band gap is calculated by extrapolating a straight line to the abscissa axis. Figure 4b shows the plot of the (αhν)^1/2^ versus Eg. The band gap of the produced TiO_2_ is estimated to be 3.15 eV, which is close to the theoretical band gap (3.2 eV) of anatase TiO_2_ powder.

**Figure 5:**
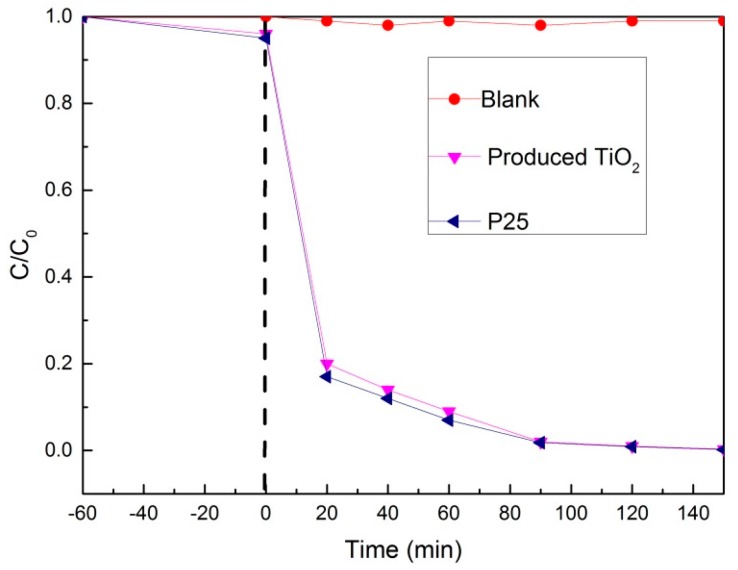
Photocatalytic decomposition of methylene blue by the produced TiO_2_ using acetylacetone as the hydrolysis control agent.

The photocatalytic performance of the TiO_2_ obtained by the calcination of TiO_2_ sol was evaluated by decomposition of an aqueous methylene blue solution under UV-light illumination (Figure 5). To achieve the adsorption equilibrium, the solution including methylene blue and the photocatalyst was stirred in dark for 60 min without UV light irradiation. A blank experiment without photocatalyst was also done under the same conditions. The blank experiment shows that during the whole process, the concentration of methylene blue solution remained almost the same, which means that methylene blue was quite stable under illumination.

From Figure 5, it can be seen clearly that the produced TiO_2_ had an obvious photocatalytic effect. Nearly 90% of the methylene blue was degraded at 40 min and almost all of it was degraded in 90 min. The produced TiO_2_ had similar photocatalytic degradation ability compared with P25. The excellent photocatalytic degradation ability of the produced TiO_2_ will be a great help to the self-cleaning performance of the TiO_2_ film.

### 3.4. The Morphology of TiO_2_ Thin Film and Its Self-Cleaning Property

#### 3.4.1. The Morphology of TiO_2_ Thin Film

**Figure 6:**
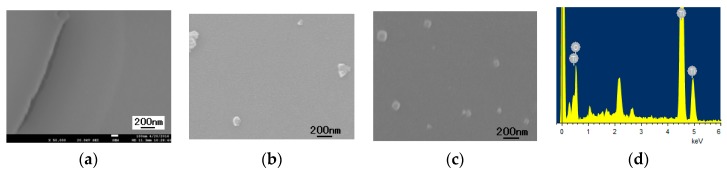
SEM images of the TiO_2_ thin film formed using acetylacetone as the hydrolysis control agent (**a**) aging time 0 h; (**b**) aging time 6 h; (**c**) aging time 48 h; (**d**) The EDS of the thin films.

Figure 6 shows the SEM images and Energy Dispersive Spectroscopy (EDS) analysis of the TiO_2_ thin film by using acetylacetone as the hydrolysis control agent at different aging times. The TiO_2_ thin film was quite compact with no aging treatment. As the aging time increased to 6 h and 48 h, a small number of particles with grain size less than 200 nm existed on the TiO_2_ film. The majority of the film was still compact and continuous. The EDS analysis shows that the tiny particles on the surface were TiO_2_. The results above show that the sol produced using acetylacetone as the hydrolysis control agent can be used to make TiO_2_ thin films.

#### 3.4.2. The Hydrophilicity and Self-Cleaning Property of the TiO_2_ Thin Film

Supplementary Materials Figure S2 shows the water contact angles of the TiO_2_ thin films formed by using TiO_2_ sol with acetylacetone as the hydrolysis control agent followed by 30 min ultraviolet illumination. When the calcination temperature was 500 °C, the water contact angle of the thin film was almost 0°, showing an extraordinary light-induced hydrophilic effect and super-hydrophilicity, which may be caused by the large amount of highly crystallized anatase TiO_2_. The film calcined at 300 °C had a relatively large contact angle because the TiO_2_ formed at this temperature was mostly amorphous and there were still some organic compounds remaining.

**Figure 7:**
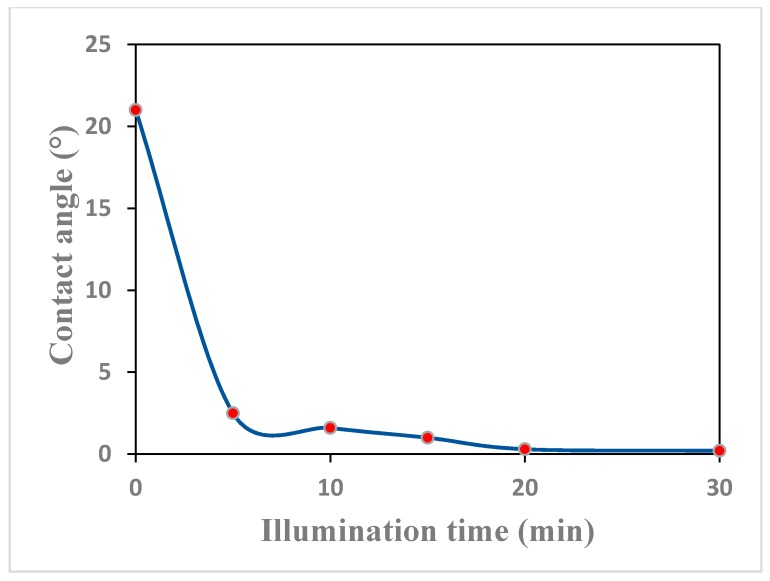
Contact angle changes of TiO_2_ film with illumination time (Acetylacetone was used as hydrolysis control agent and the calcination temperature was 500 °C).

The effect of the illumination time to the hydrophilic properties of the film was also investigated. Figure 7 shows water contact angle changes of TiO_2_ film with illumination time. Before illumination, the contact angle of TiO_2_ film was 21°, while after 5 min illumination, the contact angle was reduced to less than 5°, which showed super-hydrophilic properties. The contact angle of TiO_2_ film reduced continuously with illumination time and reached almost 0° when the illumination time was 20 min, which meant complete wetting and super hydrophilicity were realized by then. The above-mentioned light-induced super-hydrophilicity of the TiO_2_ film is an important factor in its self-cleaning ability.

#### 3.4.3. The Antifogging Property of the TiO_2_ Thin Film

**Figure 8:**
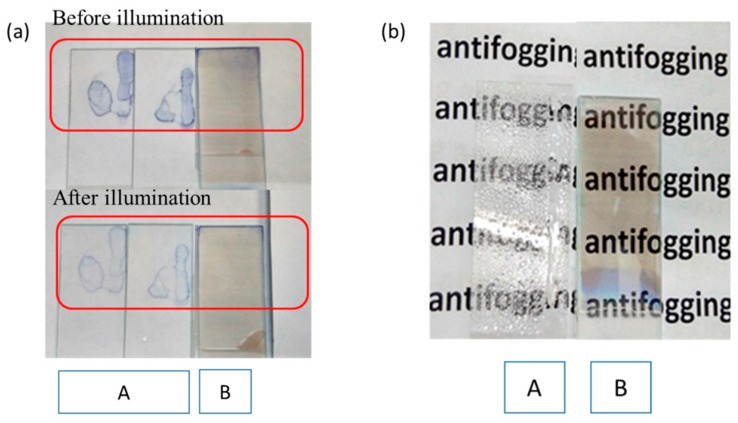
(**a**) The self-cleaning properties of TiO_2_ film; (**b**) The antifogging property of TiO_2_ film (A is the glass slides without TiO_2_ film; B is the glass slide with TiO_2_ film using acetylacetone as the hydrolysis control agent).

The self-cleaning function and antifogging property of TiO_2_ film were also tested (Figure 8). Methylene blue was used as a kind of organic pollutant to coat the surface of glass slides with or without TiO_2_ film. Compared with the glass slide itself, the TiO_2_ film before illumination had a relatively smaller water contact angle, therefore, the methylene blue coated on TiO_2_ film had a bigger coated area and the coating was more even. After illumination, the glass slides were washed slowly with water. From Figure 8a, it can be seen that the pollutant was still on the glass slides without TiO_2_ film coating with an obvious profile, while the glass slides with TiO_2_ film regained their cleanness in the center, which meant the TiO_2_ film had the self-cleaning function. The antifogging property of TiO_2_ film was also tested (Figure 8b). After illumination, the glass slides were sprayed on the surface. A large number of water droplets emerged on the surfaces of glass slides without TiO_2_ film, leading to poor vision of the background image. Instead, the glass slides with TiO_2_ film were quite clean, which showed the extraordinary antifogging properties as well as the light-induced hydrophilicity of the produced TiO_2_ film.

## 4. Conclusions

In this article, we have successfully produced TiO_2_ sol by the hydrolysis of tetrabutyl titanate using the sol-gel method. Among the three hydrolysis control agents, acetylacetone is more suitable than triethanolamine and the mixture of acetic acid and hydrochloric acid. When the calcination temperature was 500 °C, the products were in single anatase TiO_2_ phase with grain size 18–20 nm. The products after the calcination of the sol at 500 °C had strong absorption of ultraviolet light of wavelengths less than 400 nm and its band gap was calculated as 3.15 eV. It had strong photocatalytic properties and could degrade nearly 100% of methylene blue after 90 min illumination, which is similar to P25. However, it is hard to use P25 to produce TiO_2_ thin film compared with the TiO_2_ sol used in this research. The final TiO_2_ film was compact and continuous and had an obvious light-induced hydrophilic effect and super-hydrophilicity. After 30 min ultraviolet illumination, the contact angle of the obtained TiO_2_ film reached almost 0°. It also had a strong anti-fogging capability and excellent self-cleaning ability, making it suitable for products related to our daily lives.

## Figures and Tables

**Table 1:** The color and status of TiO_2_ sol with different aging times.

Hydrolysis Control Agent	0 h	6 h	18 h	48 h
acetylacetone	Brown	Brown	Brown	Brown
triethanolamine	Colorless	Pale white	Pale white	Some precipitation
acetic acid and hydrochloric acid mixture	Light brown	Light brown	Light brown	Light brown

## Supplementary Materials

The following are available online at http://www.mdpi.com/1996-1944/11/3/450/s1. Figure S1: SEM images of the TiO_2_ powders produced by using acetylacetone as hydrolysis control agent and (a) dried at 100 °C; (b) calcined at 300 °C; (c) calcined at 400 °C; (d) calcined at 600 °C. Figure S2: Contact angle of TiO_2_ film by using acetylacetone as the hydrolysis control agent, calcined at different temperatures and ultraviolet irradiated for 30 min.
