# Supplementary material for: The Preparation of TiO2 Film by the Sol-Gel Method and Evaluation of Its Self-Cleaning Property
Source: Materials (Basel). 2018 Mar 19;11(3):450. doi: 10.3390/ma11030450 (PMC5873029; doi:10.3390/ma11030450)
Supplement: Supplementary file 1 [file materials-11-00450-s001.pdf]

Supplementary materials

The Preparation of TiO_2 Film by the Sol-Gel Method and Evaluation of Its Self-Cleaning Property

Yu Liang [1,2], Sijia Sun [1], Tongrong Deng [1], Hao Ding [1,*], Wanting Chen [1] and Ying Chen [1]

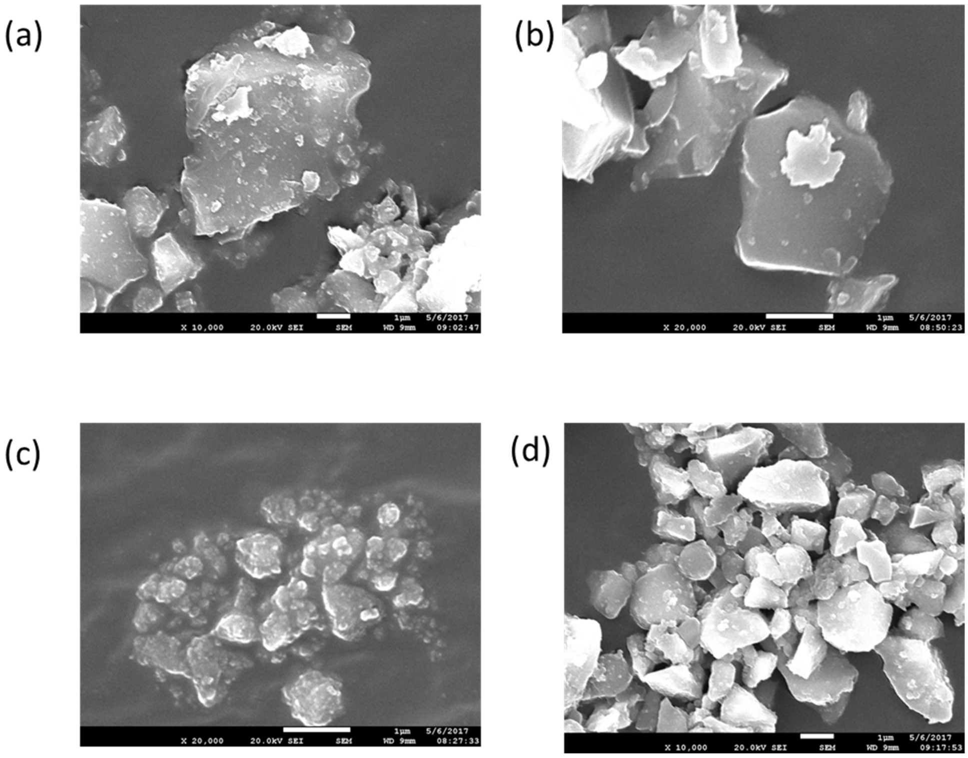

Figure S1. SEM images of the TiO_2 powders produced by using acetylacetone as hydrolysis control agent and (a) dried at 100 °C; (b) calcined at 300 °C; (c) calcined at 400 °C; (d) calcined at 600 °C.

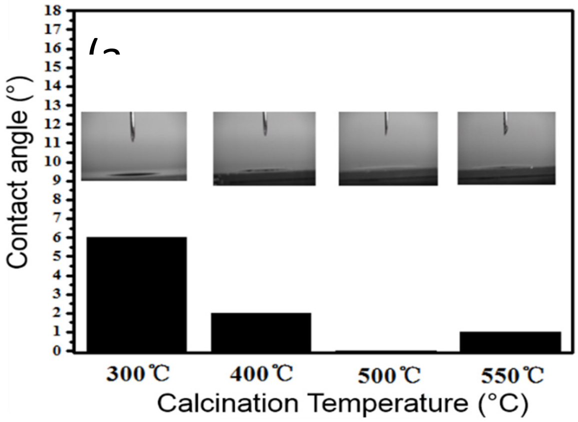

Figure S2. Contact angle of TiO_2 film by using acetylacetone as the hydrolysis control agent, calcined at different temperatures and ultraviolet irradiated for 30 min.
